# On Cold as a Means of Producing Local Insensibility

**Published:** 1848-09

**Authors:** James Arnot

**Affiliations:** Brighton


*On Cold as a means of producing Local Insensibility.* By JAMES ARNOT, M. D., Brighton.—The Lancet for July 8th contains an interesting paper by Professor Simpson, of Edinburgh, on the substitution of local anæsthetic agents for those of general action: and the publication, in the same number, of a case of sudden death from chloroform, is well calculated to draw attention to his observations. Unfortunately, however, his paper does not hold out much hope of success from any of the local means with which he has experimented.

Eight months ago, I proposed, in a work on "The Uniform Application of Heat or Cold in Various Diseases," (preface, p. viii.) to substitute local means for producing insensibility during surgical operations. One of the two measures then suggested I have proved to be effectual. I allude to the benumbing effect of cold.

There are many operations in which the only source of pain is the incision of the skin, and more in which this is the principal source. To these, this agent is especially applicable. I have, for example, after using it, made an issue by dissecting off a square inch of the skin without causing pain; and I have made setons without the patients being conscious that the skin was cut. I have little doubt that operations on the teeth, in which ether and chloroform have been so much and often so improperly used, could be rendered painless by the same means. In my "Essay on the Present State of Therapeutical Inquiry," I have spoken of cold properly or uniformly applied as an effectual remedy of one species of toothache.

In applying cold with the views under consideration, I have taken care to avoid reaction and the determination of blood to the part, by reducing the temperature very gradually. To benumb a small portion of skin, a very simple apparatus is required. A small pig's bladder, some pounded ice, and a little salt. The bladder, containing tepid water, is placed so as to cover the portion of skin to be rendered insensible; the ice is then gradually dropped in, and last of all the salt, so as to bring the temperature considerably below the freezing point. The water or dissolved ice is occasionally drawn off from the bladder, (which can be done very conveniently by having previously fixed a bit of metallic tube in it,) and when all sensation has ceased, which I have generally found to be the case after fifteen or twenty minutes, the operation should be proceeded with. A very thin or "prepared" bladder, a broad ring to keep the opening in it expanded, and a thermometer to be placed in it, would render the apparatus more complete, but they are not necessary. Of course, for other purposes, other forms of apparatus would be required.

I cannot doubt that the mode of proceeding I have related might be much improved upon. I have not paid much attention to the subject, but the above hints may be useful to those disposed to prosecute the inquiry. Perhaps, for instance, so low a degree of cold as I have mentioned is not required to produce the requisite degree of insensibility, and if pressure were conjoined with cold, so as to squeeze the blood from the part, and check the circulation, the effect would certainly be more rapid, complete, and extensive. In the little operations I have mentioned, it was unnecessary to use the precaution against injurious reaction of gradually restoring the natural temperature; and even in more important operations, when the benumbing has been of short duration, there might not be a necessity for such proceeding. Should there be such necessity, the work alluded to, in which this suggestion was first made, contains a description of means by which the temperature of a part can be completely controlled; and I shall be happy if the less important purposes of producing insensibility in operations should extend the acquaintance of the profession with these means—the most powerful, perhaps, and certainly the safest, in our profession, of subduing the most frequent and most fatal morbid conditions—inflammation. With reference to the present question, it may be observed, that by thus preventing any morbid degree of temperature, not only may reaction after low temperatures be prevented, but even that excess of inflammation which so frequently causes an unfortunate result of operations conducted in the ordinary way. *Lancet.*

MEDICAL SOCIETY OF LONDON.

*Cerebral Disease in connexion with disease of the Ear.*—Mr. Pilcher exhibited the portion of the brain of a child who had been the subject of ear disease. The child sunk immediately, from acute abscess in the left hemisphere of the brain consequent upon the ear disease. On examination after death an abscess was discovered in the lateral ventricle, communicating with the other collection of matter. The latter abscess bore the appearance of being of long standing, and was surrounded by a thick, dark, lining membrane. The question to be decided was, whether this latter abscess was an old or recent formation; if old, it most probably had existed for years. To show the way in which disease of the brain might occasionally, for a time, be masked, or the symptoms suspended, and the disease afterwards prove suddenly fatal, he related the case of a little girl who had suffered from ear disease, attended by much purulent discharge. Convulsions attended the disease, but these became much less frequent, and she recovered almost her usual health. She was so much better, indeed, that the practitioner in attendance was puzzled. The patient one day went to sleep and never awoke. An abscess was found occupying one of the lobes of the cerebellum, capable of containing four ounces of pus, and surrounded by a distinct cyst.

Mr. Hird remarked that the experiments of Mr. Mayo had seemed to establish that when pressure was exerted downwards, as in the last case related, paralysis was almost always the result; but when pressure was exerted laterally, no symptoms might present themselves. He (Mr. Hird) had seen cases in which disease of the cerebrum and upper portion of the brain was present without any, or with but few symptoms; when the disease was situated at the base of the brain, involving the medulla oblongata, symptoms were always present.

Dr. Bennett related a case of extensive disease involving a great portion of the medulla oblongata, which was not attended by symptoms of that mischief, until within a few hours of the death of the patient. The child was of healthy appearance. Squinting was the first symptom that developed itself—this was followed by convulsions. After death a tubercular mass was discovered, involving the greater potion of the medulla oblongata, and as large as a full-grown walnut.

Dr. Chowne related two cases to show that disease of the brain might exist to a considerable extent without any evidence being exhibited of the seat of the mischief. The first of these cases was that of a young woman who was admitted into Charing Cross Hospital with what might be called general illness. She had acute headache; fever, sometimes remitting, sometimes intermitting, and a peculiarity of expression of the face. She died, and an abscess, affecting one hemisphere of the brain, was discovered. In the second case the patient was a young man, admitted into the hospital "very ill," but complaining chiefly, if not entirely, of pains about the right side of the neck, like rheumatism. The neck was fomented. The countenance was peculiar, and such as he had noticed before in cases of brain disease.

He suffered from severe inflammation of the ear, suppuration came on; he lived several weeks, but had no pain in the head. A small abscess was discovered situated at the upper part of the middle lobe of the cerebrum. A yellow, thick, tenacious pus was found in the abscess. He referred to a case, which had been related to the Society some time back, of a merchant who continued to conduct an extensive and anxious business to within two or three days of his death, and on examination it was found that the anterior lobes of each hemisphere of the brain were softened to a considerable extent.

Some discussion followed on Dr. Wigan's theory of the duality of the mind.—*Lancet.*

